# Prognostic value of the NUTRIC score for 28-day mortality in patients with sepsis-associated acute respiratory distress syndrome and its comparison with other nutritional indices: a retrospective cohort study

**DOI:** 10.3389/fnut.2026.1895282

**Published:** 2026-09-03

**Authors:** Nixuan Wang, Mingxia Ji, Junfeng Wang, Chenyu Ma

**Affiliations:** 1 School of Clinical Medicine, Hangzhou Normal University, Hangzhou, Zhejiang, China; 2 Department of Critical Care Medicine, Yiwu Central Hospital, the Affiliated Yiwu Hospital of Wenzhou Medical University, Yiwu, Zhejiang, China; 3 The Third People’s Hospital Health Care Group of Cixi, Ningbo, Zhejiang, China

**Keywords:** 28-day mortality, NUTRIC score, restricted cubic spline analysis, sepsis-associated acute respiratory distress syndrome, survival analysis

## Abstract

**Background:**

Nutritional and metabolic derangements are common in patients with sepsis-associated acute respiratory distress syndrome (ARDS) and may contribute to adverse outcomes. This study evaluated the prognostic value of the Nutrition Risk in Critically Ill (NUTRIC) score for 28-day mortality in this population.

**Methods:**

A retrospective analysis was conducted on clinical data from 152 sepsis patients with ARDS admitted to the intensive care unit (ICU) at Yiwu Central Hospital between December 2023 and January 2025. Associations between NUTRIC score and 28-day mortality were examined using multivariable logistic and Cox regression. Restricted cubic spline (RCS) analysis assessed dose–response relationship. Discrimination, calibration, and clinical utility were evaluated using receiver operating characteristic (ROC) curves, calibration analysis, Brier score, and decision curve analysis (DCA). Internal validation used 1,000 bootstrap resamples. Subgroup, interaction, and Kaplan–Meier analyses were performed.

**Results:**

The 28-day mortality rate was 34.2% (52/152). After multivariable adjustment, each 1-point increase in NUTRIC score was associated with higher odds of mortality (odds ratio, 1.65; 95% CI, 1.34–2.02; *p* < 0.001) and a higher mortality hazard (hazard ratio, 1.41; 95% CI, 1.24–1.61; *p* < 0.001). Quartile analysis demonstrated a significant trend (*p* for trend < 0.001). RCS analysis indicated an approximately linear positive association (*p* for nonlinearity = 0.764), robust to alternative knot placements. The NUTRIC score showed moderate discriminative (AUC = 0.743, bootstrap 95% CI, 0.661–0.827) and performed better than mNUTRIC (AUC = 0.712, DeLong *p* = 0.003). Compared with mNUTRIC, NUTRIC improved reclassification and discrimination (net reclassification improvement, 0.219; integrated discrimination improvement, 0.044; both *p* < 0.001). Calibration was satisfactory (Brier score, 0.194; slope, 1.018; intercept, 0.020). Decision curve analysis showed net benefit at threshold probabilities of 11–60%. A significant interaction was found for septic shock (*p* for interaction = 0.038). Patients with NUTRIC scores ≥6 had lower cumulative survival (log-rank *p* < 0.001).

**Conclusion:**

In patients with sepsis-associated ARDS, higher NUTRIC scores were independently associated with increased 28-day mortality. The NUTRIC score may serve as a practical tool for early risk stratification and integrated assessment of nutritional and disease burden in critically ill patients with sepsis-associated ARDS.

## 1 Introduction

Acute respiratory distress syndrome (ARDS) is a severe form of acute inflammatory lung injury, commonly triggered by infection, trauma, shock, or other systemic insults, and is characterized by diffuse alveolar damage, increased pulmonary vascular permeability, non-cardiogenic pulmonary edema, and refractory hypoxemia (1). In 2016, approximately 10.4% of patients in intensive care units (ICUs) were diagnosed with ARDS, highlighting its substantial burden in critical care practice (2). Sepsis-associated ARDS is one of the most common and clinically important ARDS subtypes, with previous studies suggesting that approximately 30 to 40% of sepsis patients may develop ARDS (3, 4). Compared with other ARDS phenotypes, patients with sepsis-associated ARDS often present with more pronounced systemic inflammatory, greater organ dysfunction, prolonged ventilatory support, and a higher risk of death. The lungs are not only the primary organ for gas exchange but also an important immune interface; therefore, it is frequently involved early during the progression of sepsis (5, 6).

Sepsis-associated ARDS is closely linked to profound metabolic and nutritional disturbances. In patients with sepsis, activation of inflammatory cascades, endocrine stress responses, and gastrointestinal dysfunction may promote hypercatabolism and increase the risk of nutritional deterioration (7, 8). ARDS further aggravates this process through impaired gas exchange, tissue hypoxia, increased work of breathing, and the metabolic demands associated with lung injury repair and respiratory muscle recovery In addition, sedatives, analgesics, vasopressors, and mechanical ventilation may impair gastrointestinal motility and contribute to enteral feeding intolerance. Inflammation, immobilization, inadequate nutritional intake, and persistent catabolism may jointly contribute to intensive care unit-acquired weakness, which can prolong mechanical ventilation and delay functional recovery (9). These interacting mechanisms create a sustained state of metabolic stress and increased nutritional requirements, making nutritional risk particularly relevant in patients with sepsis-associated ARDS.

Nutritional therapy is an important component of critical care management. In patients exposed to severe physiological stress, appropriate nutritional support may help attenuate excessive catabolism, maintain intestinal barrier function, modulate immune and inflammatory responses, and support organ recovery (10). However, not all critically ill patients benefit equally from aggressive nutritional interventions. Therefore, nutritional risk assessment is essential for identifying patients who are more likely to benefit from targeted nutritional support and for informing individualized nutritional strategies (11). The Nutrition Risk in Critically ill (NUTRIC) score was developed specifically for ICU patients to identify those at high nutritional risk and at increased risk of adverse outcomes potentially modifiable by nutritional therapy. The original NUTRIC score incorporates six variables: age, Acute Physiology and Chronic Health Evaluation II (APACHE II) score, Sequential Organ Failure Assessment (SOFA) score, number of comorbidities, days from hospital admission to ICU admission, and interleukin-6 (IL-6) level (12–14). By integrating disease severity, inflammatory burden, comorbidity, and delayed ICU admission, the NUTRIC score may be particularly suitable for risk stratification in critically ill patients with sepsis-associated ARDS.

The hypercatabolic state induced by sepsis and the refractory hypoxemia associated with ARDS are both closely related to poor short-term outcomes. Nevertheless, evidence specifically evaluating the prognostic value of the NUTRIC score in patients with sepsis-related ARDS remains limited. Given the combined burden of systemic inflammation, organ dysfunction, respiratory failure, and nutritional risk in this population, clarifying the relationship between NUTRIC score and mortality may provide clinically useful information for early risk stratification. Therefore, this single-center retrospective cohort study aimed to evaluate the predictive value of the NUTRIC score for 28-day mortality in patients with sepsis-associated ARDS. The findings may help refine prognostic assessment and provide a basis for future studies exploring individualized nutritional strategies in this high-risk population.

## 2 Materials and methods

### 2.1 Criteria for population selection

In this retrospective observational study, we reviewed the medical records of adult patients with sepsis-associated ARDS admitted to the ICU of Yiwu Central Hospital between December 2023 and January 2025. Patients were eligible if they were aged 18 years or older, met the diagnosis of sepsis and ARDS, and had an ICU stay of at least 24 h. Sepsis was defined according to the Sepsis-3 criteria as life-threatening organ dysfunction caused by a dysregulated host response to infection, with organ dysfunction operationalized as an acute increase in the Sequential Organ Failure Assessment (SOFA) score of 2 points or more. ARDS was diagnosed according to the Berlin definition, based on acute onset of hypoxemia, bilateral pulmonary opacities on chest imaging, respiratory failure not fully explained by cardiac failure or fluid overload, and oxygenation impairment assessed by the PaO_2_/FiO_2_ ratio under positive end-expiratory pressure or continuous positive airway pressure.

**Figure 1:**
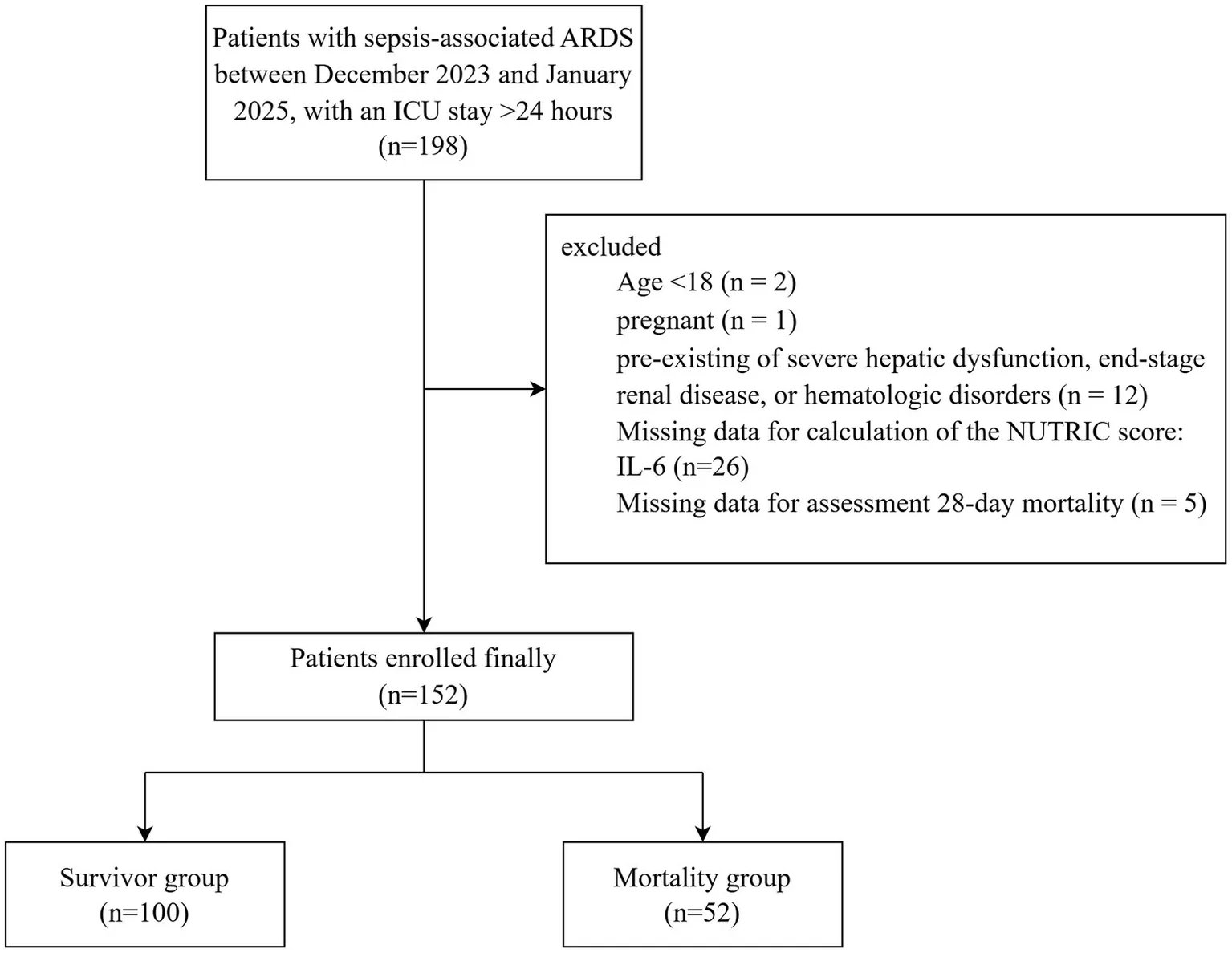
Flowchart of patient enrollment and exclusion.

Patients were excluded if they were younger than 18 years, had an ICU stay shorter than 24 h, had incomplete clinical data required for calculation of the NUTRIC score or assessment of 28-day mortality, or were pregnant. Patients with pre-existing severe hepatic dysfunction, end-stage renal disease, or hematologic disorders such as severe anemia, leukemia, or bone marrow dysfunction, because these conditions could substantially affect inflammatory, metabolic, and nutritional indicators. The Ethics Committee of Yiwu Central Hospital, No. K2026-IRB-083 approved this research. The patient selection process is detailed in Figure 1.

### 2.2 Data extraction

Data were extracted from the electronic medical records of patients admitted to the ICU at Yiwu Central Hospital. The index time point was defined as ICU admission. The primary outcome was all-cause mortality within 28 days of ICU admission. Baseline demographic and clinical variables included age, sex, primary diagnosis, use of vasopressors, length of ICU stay, duration of invasive mechanical ventilation, and survival status within 28 days. Vital signs and level of consciousness at ICU admission were recorded, including mean arterial pressure (MAP), pulse rate, and body temperature. Arterial blood gas analysis, electrolyte panels, and lactate levels were also collected.

Comorbidities include a history of hypertension, diabetes mellitus, and cancer. Laboratory parameters obtained within the first 24 h after ICU admission included platelet count, high-sensitivity C-reactive protein (CRP), lymphocyte count (Lym), procalcitonin, albumin, aspartate aminotransferase (AST), creatinine, triglycerides (TG), alkaline phosphatase (ALP), and IL-6. Clinical scores assessed within the first 24 h after ICU admission included the NUTRIC score, modified NUTRIC score, Prognostic Nutritional Index (PNI), Controlling Nutritional Status (CONUT) score, SOFA score, and the APACHE II score.

The original NUTRIC score was calculated using six variables: age, number of comorbidities, numbers of days from hospital admission to ICU admission, APACHE II, SOFA scores, and IL-6 level. The total score ranges from 0 to 10, with higher scores indicating greater nutritional risk and higher risk of adverse clinical outcomes. The modified NUTRIC score was calculated by excluding IL-6 from the original score. PNI and CONUT scores were calculated according to previously established formulas using routinely available laboratory indicators.

### 2.3 Statistical analysis

Patients were divided into the survivor group and non-survivor group according to 28-day survival status after ICU admission. Baseline characteristics were compared between the two groups. Continuous variables with a normal distribution were expressed as mean ± standard deviation (x ± s), and comparisons between the two groups were performed using an independent samples t-test. When the data followed a skewed distribution, the median (interquartile range) [M (Q1, Q3)] was used to represent the data, and the non-parametric Mann–Whitney U test was applied for group comparisons. Categorical variables were expressed as number and percentage and compared using the chi-square test or Fisher’s exact test, as appropriate. Missing data for covariates with <30% missingness were imputed via multiple imputation by chained equations.

Multivariable logistic regression analysis was performed to evaluate the association between the NUTRIC score and 28-day mortality. The NUTRIC score was analyzed both as a continuous variable and as a categorical variable according to quartiles. Odds ratios (ORs) and 95% confidence intervals (CIs) were calculated. Covariates were selected based on clinical relevance, prior literature and baseline differences between groups, while avoiding inappropriate adjustment for variables that were components of the NUTRIC score. Multicollinearity among independent variables was examined using the variance inflation factor (VIF). A VIF value greater than 5 was considered indicative of problematic collinearity. Four regression models were constructed with progressive adjustment to assess the robustness of the association. A test for linear trend across quartiles was performed by entering the median NUTRIC score value of each quartile as a continuous variable. As a sensitivity analysis, multivariable Cox proportional hazards regression was conducted using the same four models and quartile-based approach, with the proportional hazards assumption tested using Schoenfeld residuals.

Restricted Cubic Spline (RCS) analysis with four knots located at the 5th, 35th, 65th, and 95th percentiles of the NUTRIC score distribution was used to examine the dose–response relationship between the NUTRIC score and the odds of 28-day mortality and to assess potential nonlinearity. Sensitivity of the results to knot placement was evaluated by repeating the analysis using three knots (at the 10th, 50th, and 90th percentiles) and five knots (at the 5th, 27.5th, 50th, 72.5th, and 95th percentiles).

Receiver Operating Characteristic (ROC) curve analysis was performed to evaluate the discriminative ability of the NUTRIC score for predicting 28-day mortality. Bootstrap internal validation with 1,000 resamples was performed to obtain the bias-corrected area under the ROC curve (AUC) and its 95% confidence interval using the bootstrap percentile method. The optimal cutoff value was determined using the Youden index, and the corresponding sensitivity and specificity were calculated. Cutoff stability was evaluated by recalculating the Youden-optimal cutoff in each of the 1,000 bootstrap samples. Pairwise comparisons of AUCs between the NUTRIC score and the mNUTRIC, PNI, and CONUT scores were performed using DeLong’s test. Incremental prognostic value was assessed using categorical and category-free net reclassification improvement (NRI) and integrated discrimination improvement (IDI). Calibration was evaluated using the calibration plot, the Brier score, and bootstrap-derived calibration metrics.

Decision Curve Analysis (DCA) was used to evaluate the potential clinical net benefit of the NUTRIC score across a range of threshold probabilities. The net benefit of the NUTRIC score was compared with those of SOFA, APACHE II, and the default strategies of treating all or treating no patients.

Subgroup analyses were performed for septic shock, ARDS severity, site of infection, and vasopressor use. Interactions were tested using the likelihood ratio test. No adjustment for multiplicity was applied, as these analyses were exploratory.

For survival analysis, patients were stratified into high-risk and low-risk groups according to the optimal cutoff value of the NUTRIC score identified by ROC analysis. Kaplan–Meier curves were generated to compare cumulative survival probabilities between groups, and differences were assessed using the log-rank test. All statistical analyses were performed using R software version 4.6.1 and SPSS software version 27.0. All tests were two-sided, and a *p* value < 0.05 was considered statistically significant.

## 3 Results

### 3.1 Baseline characteristics

A total of 152 patients with sepsis-associated ARDS were included in this study. Among them, 100 patients (65.8%) survived to day 28 after ICU admission (survival group), whereas 52 patients (34.2%) died within 28 days (mortality group).

Compared with survivors, non-survivors were more likely to require vasopressors, had more sepsis shock and higher lactate, procalcitonin, creatinine, aspartate aminotransferase, IL-6, SOFA, APACHE II, NUTRIC, and CONUT score, whereas mean arterial pressure, platelet count, albumin, triglyceride levels, and PNI were lower in the non-survivor group (all *p* < 0.05). No statistically significant differences were observed between the two groups in age, sex, diabetes mellitus, hypertension, cancer, potassium levels, body temperature, pulse rate, high-sensitivity C-reactive protein, lymphocyte count, or alkaline phosphatase level (all *p* > 0.05; Table 1).

**Table 1:** Baseline characteristics of survivors and non-survivors.

Variables	Total (*n* = 152)	Survival (*n* = 100)	Death (*n* = 52)	*p*
Age, year^2^	76.50 (63.75, 83.00)	76.00 (60.00, 83.00)	77.50 (66.75, 84.00)	0.413
Sex, *n* (%)^3^				0.854
Female	60 (39.47)	40 (40.00)	20 (38.46)	
Male	92 (60.53)	60 (60.00)	32 (61.54)	
Severity, *n* (%)^3^				0.471
Mild	69 (45.39)	48 (48.00)	21 (40.38)	
Moderate	68 (44.74)	44 (44.00)	24 (46.15)	
Severe	15 (9.87)	8 (8.00)	7 (13.46)	
Infection site, *n* (%)^3^				0.077
Pulmonary	88 (57.89)	63 (63.00)	25 (48.08)	
Extrapulmonary	64 (42.11)	37 (37.00)	27 (51.92)	
Sepsisshock, *n* (%)^3^	59 (38.82)	33 (33.00)	26 (50.00)	0.041
Vasopressors, *n* (%)^3^	45 (29.61)	23 (23.00)	22 (42.31)	0.013
Diabetes, *n* (%)^3^	52 (34.21)	34 (34.00)	18 (34.62)	0.940
Hypertension, *n* (%)^3^	83 (54.61)	52 (52.00)	31 (59.62)	0.371
Cancer, *n* (%)^3^	20 (13.16)	15 (15.00)	5 (9.62)	0.351
Temperature, °C^1^	37.51 ± 0.93	37.53 ± 0.89	37.48 ± 1.01	0.748
Pulse rate, bpm^2^	103.00 (76.50, 120.00)	101.50 (75.00, 118.00)	109.50 (80.00, 124.75)	0.077
MAP, mmHg^2^	68.50 (59.00, 84.25)	71.00 (64.00, 87.25)	60.00 (55.00, 74.00)	<0.001
Potassium, mmol/L^2^	3.90 (3.60, 4.50)	3.90 (3.60, 4.43)	3.95 (3.58, 4.60)	0.887
Lactate, mmol/L^2^	1.90 (1.20, 3.62)	1.60 (1.10, 2.40)	3.15 (1.67, 5.17)	<0.001
Lymphocyte count, 10^9^/L^2^	0.61 (0.36, 1.16)	0.65 (0.40, 1.19)	0.49 (0.24, 1.05)	0.057
Platelet count, 10^9^/L^2^	156.00 (103.00, 210.25)	180.00 (109.50, 226.00)	129.50 (87.75, 172.75)	0.001
CRP, mg/L^2^	87.96 (31.67, 154.84)	75.67 (30.05, 148.93)	98.80 (47.79, 159.05)	0.342
Procalcitonin, ng/ml^2^	3.76 (1.17, 17.79)	2.21 (0.89, 10.79)	10.28 (2.60, 46.15)	<0.001
Albumin, g/L^1^	28.26 ± 4.88	28.89 ± 4.45	27.03 ± 5.45	0.025
AST, U/L^2^	43.50 (23.75, 113.50)	36.50 (20, 82.50)	57.50 (32.75, 295.75)	0.002
ALP, U/L^2^	66.50 (52.75, 91.25)	68.00 (53.75, 94.25)	64.50 (49, 85.50)	0.229
Triglycerides, mmol/L^2^	1.00 (0.79, 1.69)	1.15 (0.83, 1.93)	0.93 (0.68, 1.42)	0.038
Creatinine, μmol/L^2^	126.60 (81.80, 251.68)	105.15 (69.05, 209.95)	210.25 (110.92, 268.60)	<0.001
Interleukin-6, pg./ml^2^	134.09 (31.85, 963.38)	76.12 (18.39, 271.75)	1068.01 (88.19, 5608.14)	<0.001
SOFA^2^	8.00 (6.00, 10.00)	7.00 (5.00, 9.00)	10.00 (7.75, 14.25)	<0.001
APACHE II^1^	21.58 ± 7.65	19.43 ± 6.32	25.71 ± 8.31	<0.001
NUTRIC^2^	5.00 (3.75, 7.00)	4.00 (3.00, 6.00)	6.00 (5.00, 8.00)	<0.001
PNI^2^	31.73 (28.34, 37.30)	32.30 (29.69, 37.39)	29.55 (25.50, 36.41)	0.014
CONUT^2^	8.00 (6.00, 10.00)	7.00 (5.75, 9.00)	9.00 (7.00, 11.00)	0.002

MAP, mean arterial pressure; CRP, C-reactive protein; AST, aspartate aminotransferase; ALP, alkaline phosphatase; SOFA, Sequential Organ Failure Assessment; APACHE II, Acute Physiology and Chronic Health Evaluation II; NUTRIC, Nutrition Risk in the Critically Ill; PNI, Prognostic Nutritional Index; CONUT, Controlling Nutritional Status. Data are presented as mean ± standard deviation, median (interquartile range), or *n* (%), as appropriate. *p* values were calculated using the Student’s *t* test^1^, Mann–Whitney U test^2^, or Chi-square test^3^, as appropriate.

### 3.2 Association between NUTRIC score and 28-day mortality

The association between the NUTRIC score and 28-day mortality was examined using four logistic regression models with progressive adjustment (Table 2). All VIF values were well below the threshold of 5, ranging from 1.010 to 1.079, indicating that multicollinearity was not a concern in the regression model. The crude model (Model 1) was sequentially refined by adjusting for sex (Model 2), then for medical history and comorbidities, including hypertension and cancer (Model 3), and finally for laboratory parameters (Lym and ALP) in Model 4. In Model 1, the NUTRIC score was significantly positively correlated with 28-day mortality in patients with sepsis complicated by ARDS (OR = 1.54, 95% CI: 1.28–1.85, *p* < 0.001). Among all models, Model 4 showed the strongest association, with an OR of 1.65 (95% CI: 1.34–2.02, *p* < 0.001), followed by Model 3 (OR = 1.59, 95% CI: 1.31–1.93, *p* < 0.001), Model 2 (OR = 1.55, 95% CI: 1.29–1.87, *p* < 0.001), and Model 1 (OR = 1.54, 95% CI: 1.28–1.85, *p* < 0.001). In the final adjusted model, each one-unit increase in the NUTRIC score was associated with a 65% higher odds of 28-day mortality (OR = 1.65), indicating that the NUTRIC score is an independent risk factor for mortality in sepsis patients with ARDS.

**Table 2:** Multivariate logistic regression results for multiple models.

Variables	Model 1		Model 2		Model 3		Model 4	
	OR (95%CI)	*p*	OR (95%CI)	*p*	OR (95%CI)	*p*	OR (95%CI)	*p*
NUTRIC	1.54 (1.28 ~ 1.85)	<0.001	1.55 (1.29 ~ 1.87)	<0.001	1.59 (1.31 ~ 1.93)	<0.001	1.65 (1.34 ~ 2.02)	<0.001
NUTRIC1	1.00 (Reference)		1.00 (Reference)		1.00 (Reference)		1.00 (Reference)	
NUTRIC2	2.36 (0.56 ~ 9.90)	0.240	2.41 (0.57 ~ 10.15)	0.229	2.46 (0.58 ~ 10.43)	0.221	2.40 (0.56 ~ 10.22)	0.236
NUTRIC3	4.94 (1.50 ~ 16.19)	0.008	5.08 (1.54 ~ 16.74)	0.008	4.98 (1.50 ~ 16.55)	0.009	5.43 (1.61 ~ 18.29)	0.006
NUTRIC4	12.50 (3.74 ~ 41.72)	<0.001	13.08 (3.88 ~ 44.09)	<0.001	14.68 (4.26 ~ 50.59)	<0.001	16.69 (4.74 ~ 58.80)	<0.001
Trend test	1.56 (1.29 ~ 1.89)	<0.001	1.57 (1.29 ~ 1.91)	<0.001	1.62 (1.32 ~ 1.98)	<0.001	1.66 (1.35 ~ 2.05)	<0.001

OR, Odds ratio; CI, Confidence interval. Model 1: Crude. Model 2: Adjusted for sex. Model 3: Adjusted for sex, hypertension and cancer. Model 4: Adjusted for sex, hypertension, cancer, Lym, and ALP.

Cox regression yielded consistent findings (Table 3). The hazard of 28-day mortality rose with each unit increase in the NUTRIC score, with hazard ratios in Model 1 (HR = 1.38, 95% CI: 1.21–1.57, *p* < 0.001), Model 2 (HR = 1.38, 95% CI: 1.21–1.57, *p* < 0.001), Model 3 (HR = 1.41, 95% CI: 1.23–1.62, *p* < 0.001), and Model 4 (HR = 1.41, 95% CI: 1.24–1.61, *p* < 0.001). After full adjustment, each unit increase in the NUTRIC score conferred a 41% higher hazard of death within 28 days (HR = 1.41), reinforcing the conclusion that the NUTRIC score independently predicts short-term mortality. The proportional hazards assumption was not rejected for the NUTRIC score in the fully adjusted model (Schoenfeld residual test, *p* = 0.562). Graphical assessment of the scaled Schoenfeld residuals likewise showed no clear systematic variation in the NUTRIC coefficient over follow-up (Supplementary Figure S1).

**Table 3:** Multivariate cox regression results for multiple models.

Variables	Model 1		Model 2		Model 3		Model 4	
	HR (95%CI)	*p*	HR (95%CI)	*p*	HR (95%CI)	*p*	HR (95%CI)	*p*
NUTRIC	1.38 (1.21 ~ 1.57)	<0.001	1.38 (1.21 ~ 1.57)	<0.001	1.41 (1.23 ~ 1.62)	<0.001	1.41 (1.24 ~ 1.61)	<0.001
NUTRIC1	1.00 (Reference)		1.00 (Reference)		1.00 (Reference)		1.00 (Reference)	
NUTRIC2	2.22 (0.60 ~ 8.27)	0.234	2.22 (0.60 ~ 8.27)	0.234	2.35 (0.63 ~ 8.78)	0.203	2.33 (0.62 ~ 8.70)	0.209
NUTRIC3	3.78 (1.28 ~ 11.17)	0.016	3.78 (1.28 ~ 11.17)	0.016	3.83 (1.29 ~ 11.41)	0.016	4.07 (1.36 ~ 12.17)	0.012
NUTRIC4	7.73 (2.69 ~ 22.26)	<0.001	7.73 (2.69 ~ 22.26)	<0.001	8.42 (2.91 ~ 24.38)	<0.001	8.65 (2.99 ~ 25.05)	<0.001
Trend test	1.39 (1.21 ~ 1.60)	<0.001	1.39 (1.21 ~ 1.60)	<0.001	1.42 (1.23 ~ 1.63)	<0.001	1.42 (1.23 ~ 1.63)	<0.001

HR, Hazard ratio; CI, Confidence interval. Model 1: Crude. Model 2: Adjusted for sex. Model 3: Adjusted for sex, hypertension and cancer. Model 4: Adjusted for sex, hypertension, cancer, Lym, and ALP.

When the NUTRIC scores was further analyzed according to quartiles, the odds of 28-day mortality in logistic regression models and the hazards of 28-day mortality in Cox regression models increased progressively across higher NUTRIC score categories. This trend was observed in all models and remained statistically significant after full adjustment (*p* for trend < 0.001; Tables 2, 3), supporting a graded association between higher NUTRIC scores and short-term mortality.

### 3.3 RCS analysis of NUTRIC score and 28-day mortality

RCS analysis with four knots placed at the 5th, 35th, 65th, and 95th percentiles was performed to further characterize the association between the NUTRIC score and 28-day mortality. The overall association was statistically significant (*p* for overall < 0.001), whereas the test for nonlinearity was not significant (*p* for nonlinear = 0.764). These findings suggest an approximately linear positive association between the NUTRIC score and the odds of 28-day mortality.

**Figure 2:**
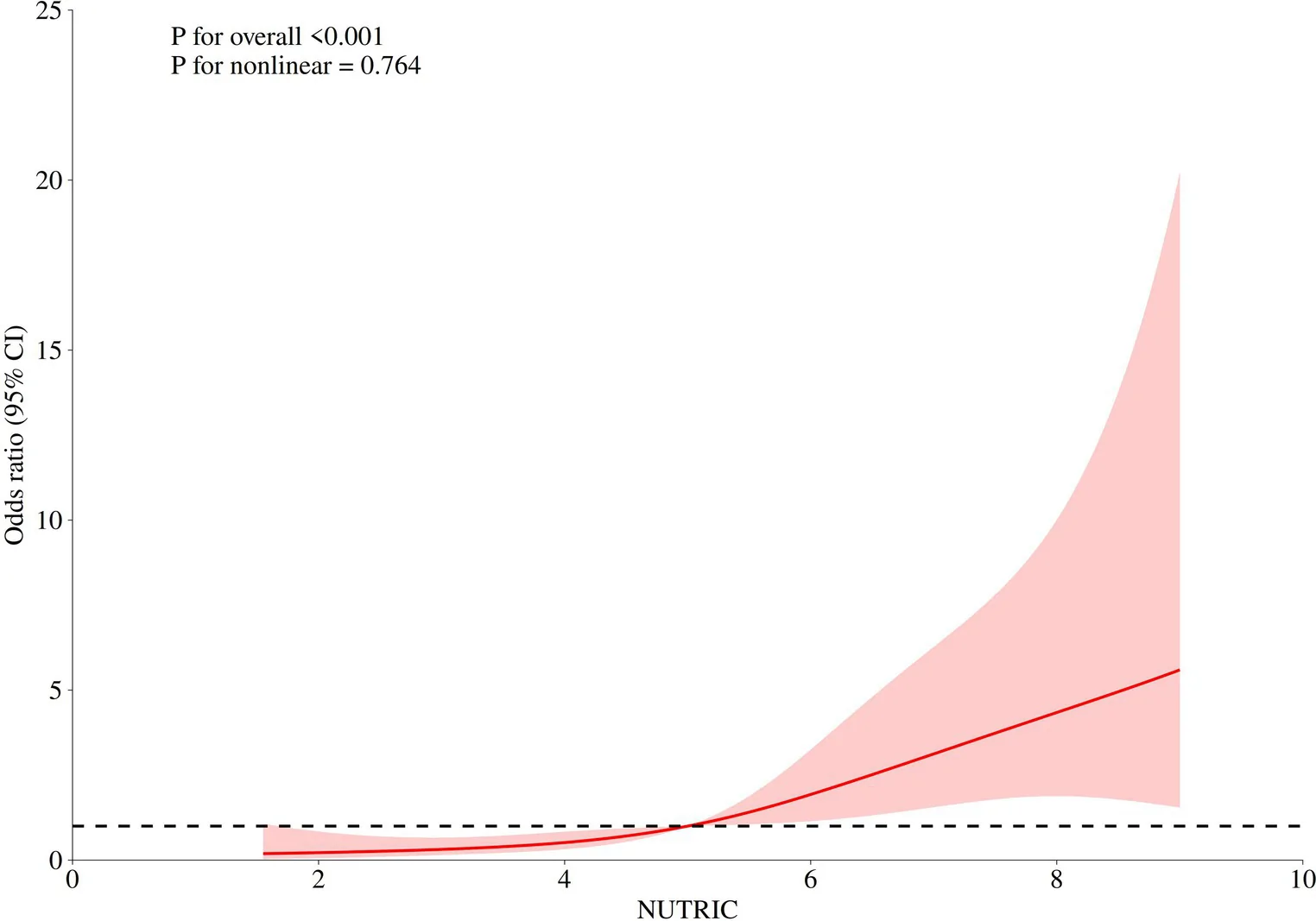
RCS analysis of NUTRIC score and 28-day mortality. CI, confidence interval; NUTRIC, Nutrition Risk in the Critically Ill.

As shown in Figure 2, the estimated odds of 28-day mortality increased gradually with increasing NUTRIC score, without clear evidence of a nonlinear threshold effect. This result supports the use of the NUTRIC score as a continuous risk stratification indicator in patients with sepsis-associated ARDS. Sensitivity analyses using three and five knots yielded similar results, confirming the robustness of the linear association to the choice of knot locations (Supplementary Figure S2).

### 3.4 Discriminative performance and internal validation

**Figure 3:**
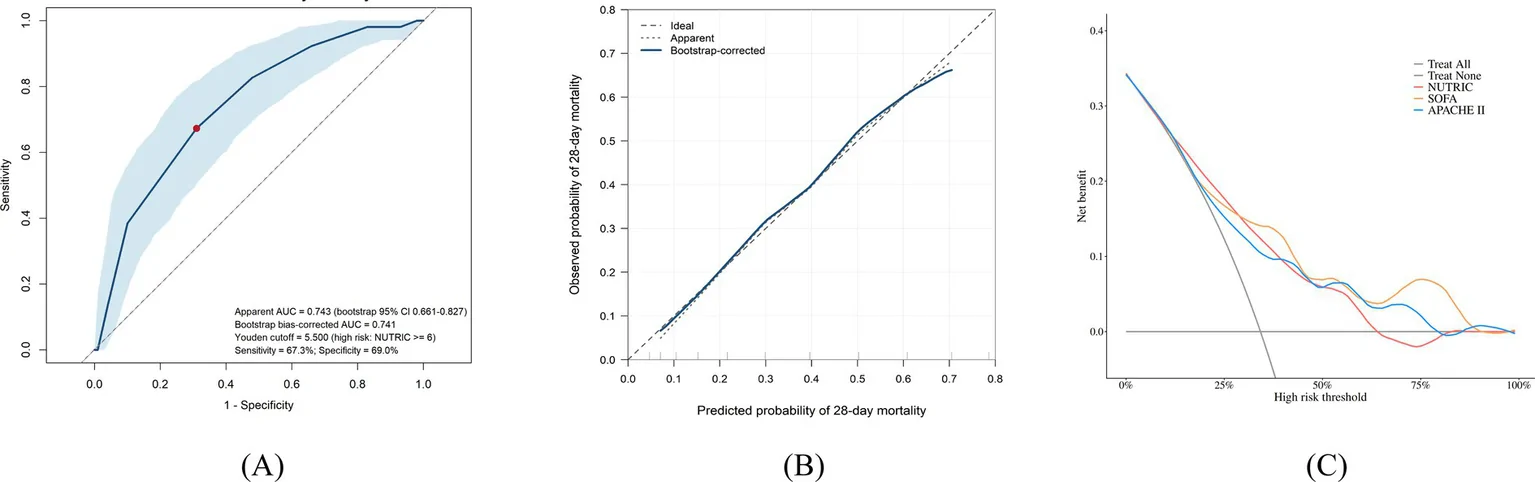
Discriminative performance, calibration, and clinical utility of the NUTRIC score for predicting 28-day mortality in patients with sepsis-associated ARDS. **(A)** Receiver operating characteristic (ROC) curve with bootstrap internal validation (1,000 resamples). **(B)** Calibration plot. The diagonal dashed line represents perfect agreement between predicted and observed probabilities. The solid line shows the calibration curve derived from 1,000 bootstrap resamples. The bias-corrected calibration slope was 1.018, the calibration intercept was 0.020, and the Brier score was 0.194. **(C)** Decision curve analysis. The net benefit of the NUTRIC score is compared with the treat-all, treat-none, SOFA, and APACHE II strategies across threshold probabilities of 0 to 60%. The NUTRIC score provided a positive net benefit from 11 to 60% threshold probability. The y-axis represents net benefit.

The NUTRIC score yielded an apparent AUC of 0.743 (bootstrap 95% CI, 0.661–0.827) for 28-day mortality. After bootstrap bias correction, the AUC was 0.741, indicating minimal bootstrap-estimated bias. The apparent Youden-optimal cutoff was 5.5, corresponding to NUTRIC ≥6, with a sensitivity of 67.3% and a specificity of 69.0% (Figure 3A). The median bootstrap-selected cutoff was 5.5 (interquartile range, 4.5–5.5; 95% percentile interval, 4.5–7.5) (Supplementary Figure S3).

**Figure 4:**
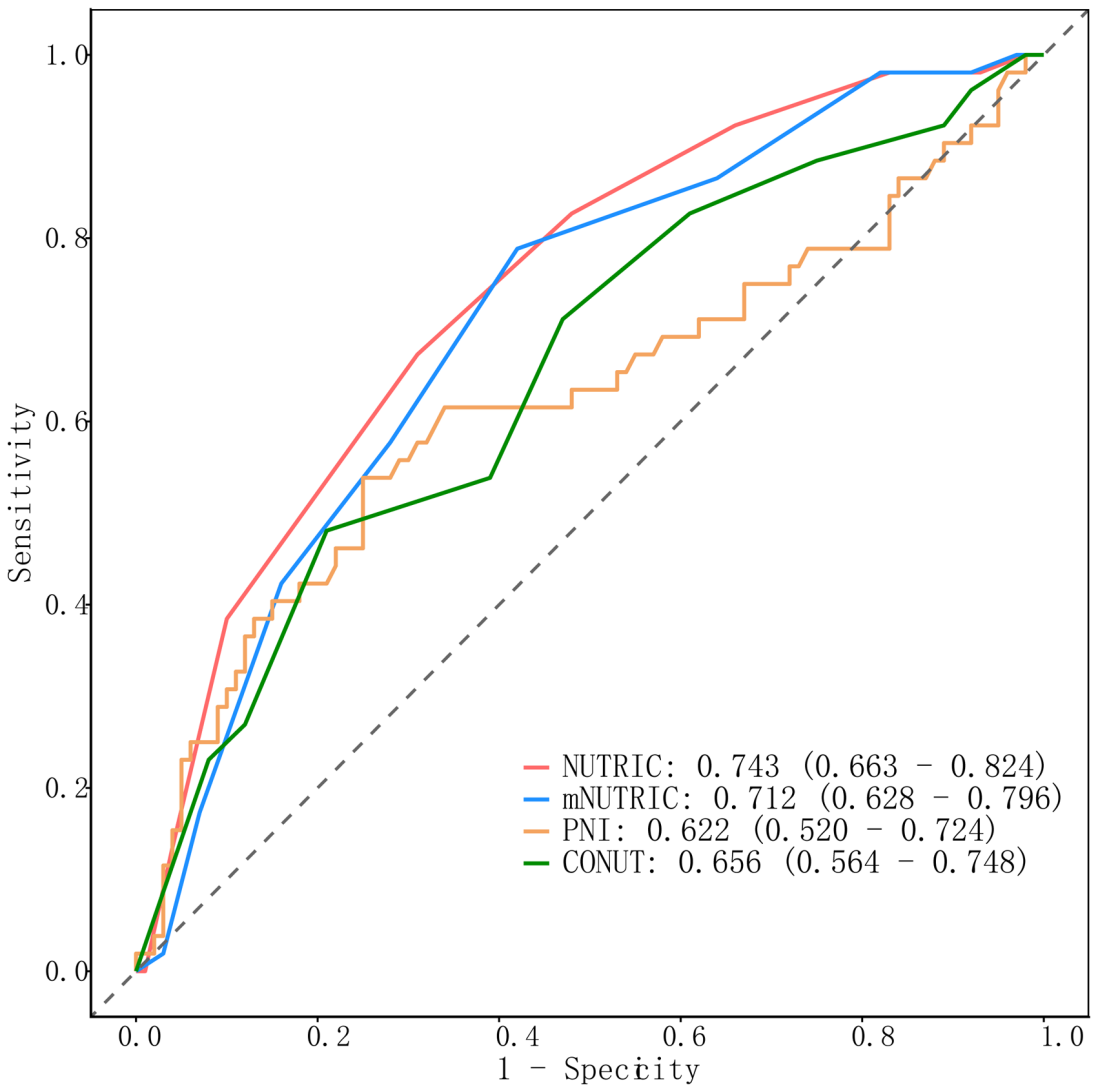
ROC for the 28-day mortality for sepsis patients with ARDS. NUTRIC, Nutrition Risk in Critically Ill Score; mNUTRIC, modified Nutrition Risk in Critically Ill Score; PNI, Prognostic Nutritional Index; CONUT, Controlling Nutritional Status Score.

The discriminative performance of the NUTRIC score (aparent AUC, 0.743; aparent 95% CI,0.662–0.824) was further compared with that of mNUTRIC, PNI, and CONUT. The corresponding apparent AUCs were 0.712 (95% CI, 0.628–0.797) for mNUTRIC, 0.622 (95% CI, 0.521–0.723) for PNI, and 0.656 (95% CI, 0.564–0.748) for CONUT (Table 4; Figure 4). Pairwise DeLong tests showed that the AUC of the NUTRIC score was significantly higher than that of mNUTRIC (*p* = 0.003). Although the NUTRIC score also had numerically higher AUCs than PNI and CONUT, these differences were not statistically significant (PNI, *p* = 0.057; CONUT, *p* = 0.128) (Table 5).

**Table 4:** The ROC curves of NUTRIC, mNUTRIC, PNI and CONUT for the prediction of 28-day mortality of all sepsis with ARDS patients.

Variables	Cut-Off	AUC	95%CI	Sensitivity	Specificity	*p* value
NUTRIC	5.5	0.743	0.662–0.824	0.673	0.69	<0.001
mNUTRIC	4.5	0.712	0.628–0.797	0.788	0.58	<0.001
PNI	29.8	0.622	0.521–0.723	0.538	0.75	0.014
CONUT	9.5	0.656	0.564–0.748	0.481	0.79	0.002

**Table 5:** DeLong test for comparison of AUCs between the NUTRIC score and other nutritional indices.

Comparison	AUC	ΔAUC (95% CI)	*Z*	*p* value
NUTRIC vs. mNUTRIC	0.743 vs. 0.712	0.031 (0.011–0.051)	2.975	0.003
NUTRIC vs. PNI	0.743 vs. 0.622	0.121 (−0.004–0.246)	1.903	0.057
NUTRIC vs. CONUT	0.743 vs. 0.656	0.087 (−0.025–0.200)	1.522	0.128

Differences in risk reclassification and discrimination were further evaluated using the net reclassification improvement (NRI) and integrated discrimination improvement (IDI). Compared with mNUTRIC, the NUTRIC score showed significant improvements in categorical reclassification (NRI, 0.219; 95% CI, 0.091–0.347; *p* < 0.001), continuous reclassification (NRI, 0.752; 95% CI, 0.440–1.065; *p* < 0.001), and discrimination (IDI, 0.044; 95% CI, 0.028–0.060; *p* < 0.001). In contrast, no statistically significant improvement was observed when the NUTRIC score was compared with SOFA (categorical NRI, −0.007; *p* = 0.942; continuous NRI, −0.215; *p* = 0.205; IDI, −0.047; *p* = 0.055) or APACHE II (categorical NRI, 0.152; *p* = 0.106; continuous NRI, 0.254; *p* = 0.134; IDI, 0.005; *p* = 0.844) (Table 6). Overall, the NUTRIC score demonstrated better discrimination and risk reclassification than mNUTRIC but did not show statistically significant improvement relative to SOFA or APACHE II in the present cohort.

**Table 6:** NRI and IDI for comparing the NUTRIC score with mNUTRIC, SOFA, and APACHE II.

Comparison	Categorical NRI (95% CI)	*p* value	Continuous NRI (95% CI)	*p* value	IDI (95% CI)	*p* value
NUTRIC vs. mNUTRIC	0.219 (0.091–0.347)	<0.001	0.752 (0.440–1.065)	<0.001	0.044 (0.028–0.060)	<0.001
NUTRIC vs. SOFA	−0.007 (−0.193–0.179)	0.942	−0.215 (−0.548–0.118)	0.205	−0.047 (−0.095–0.001)	0.055
NUTRIC vs. APACHE II	0.152 (−0.032–0.335)	0.106	0.254 (−0.078–0.586)	0.134	0.005 (−0.044–0.054)	0.844

### 3.5 Calibration plot and DCA

The calibration plot showed close agreement between the predicted probabilities and observed 28-day mortality across the evaluated risk range (Figure 3B). After internal validation with 1,000 bootstrap resamples, the bias-corrected Brier score was 0.194. The bootstrap-corrected calibration slope was 1.018, and the calibration intercept was 0.020, both close to their ideal values of 1 and 0, respectively.

DCA showed that use of the NUTRIC score provided net benefit across a range of clinically relevant threshold probabilities (11 to 60%), outperforming both the treat-all and treat-none strategies within this interval. This suggests potential utility for risk stratification in patients with sepsis-associated ARDS, Moreover, the NUTRIC score showed net benefit comparable to that of SOFA and APACHE II across most of this range (Figure 3C). However, this finding should be interpreted as evidence of potential clinical applicability rather than as proof of benefit from nutrition-guided intervention.

### 3.6 Subgroup and interaction analyses

**Figure 5:**
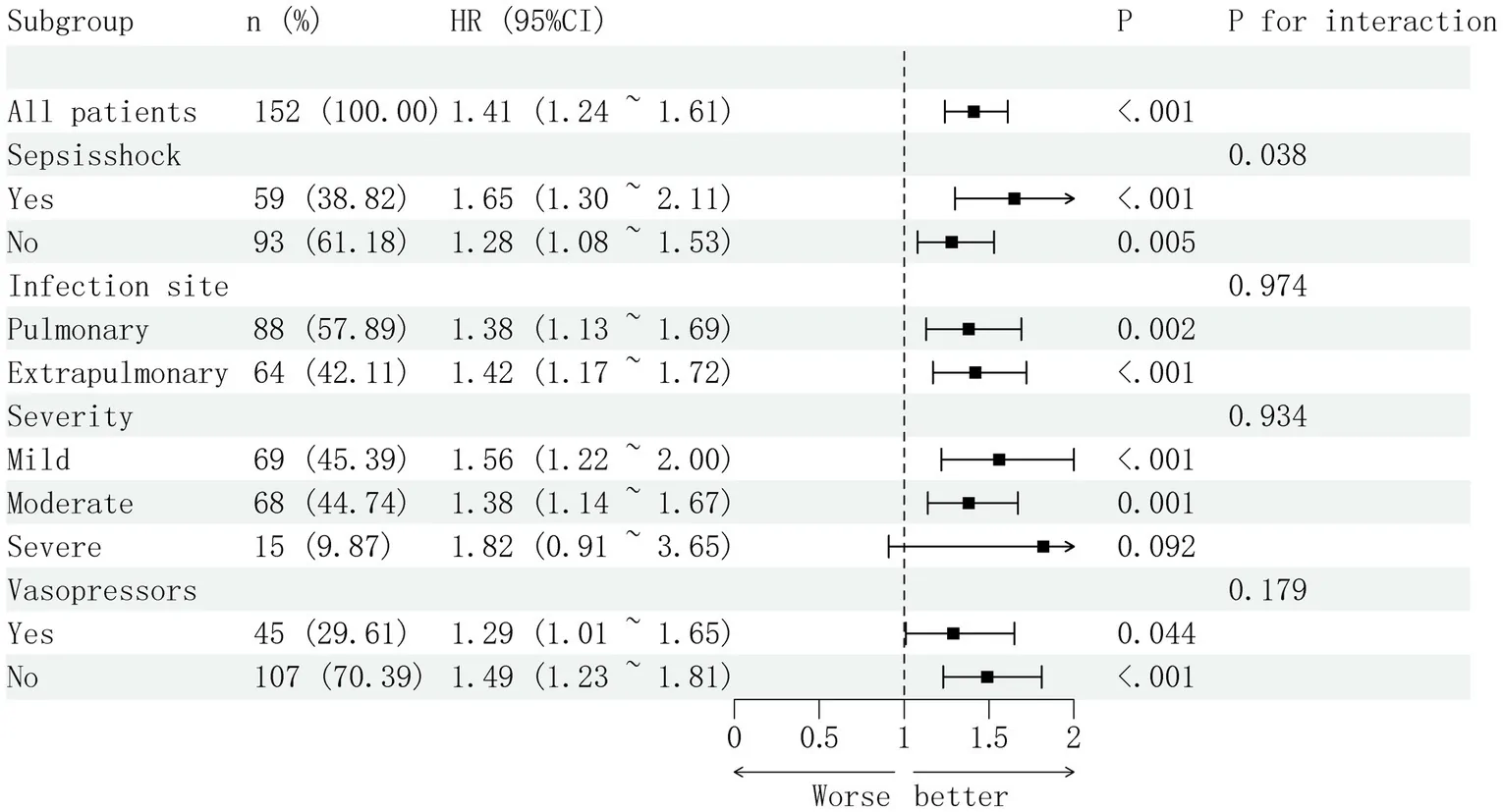
Subgroup analysis of the association between the NUTRIC score and 28-day mortality.

The multivariable-adjusted associations between the NUTRIC score and 28-day mortality across prespecified subgroups are presented in Figure 5. The direction of the associations was generally consistent with that observed in the primary analysis. A statistically significant interaction was observed between the NUTRIC score and septic shock status (*p* for interaction = 0.038). Each 1-point increase in the NUTRIC score was associated with higher hazard of 28-day mortality both in patients with septic shock (HR, 1.65; 95% CI, 1.30–2.11) and in those without septic shock (adjusted HR, 1.28; 95% CI, 1.08–1.53), with a stronger association observed in the septic shock subgroup. No statistically significant interactions were identified according to infection site (*p* for interaction = 0.974), ARDS severity (*p* for interaction = 0.934), or vasopressor use (*p* for interaction = 0.179).

### 3.7 Kaplan–Meier survival analysis according to NUTRIC score stratification

**Figure 6:**
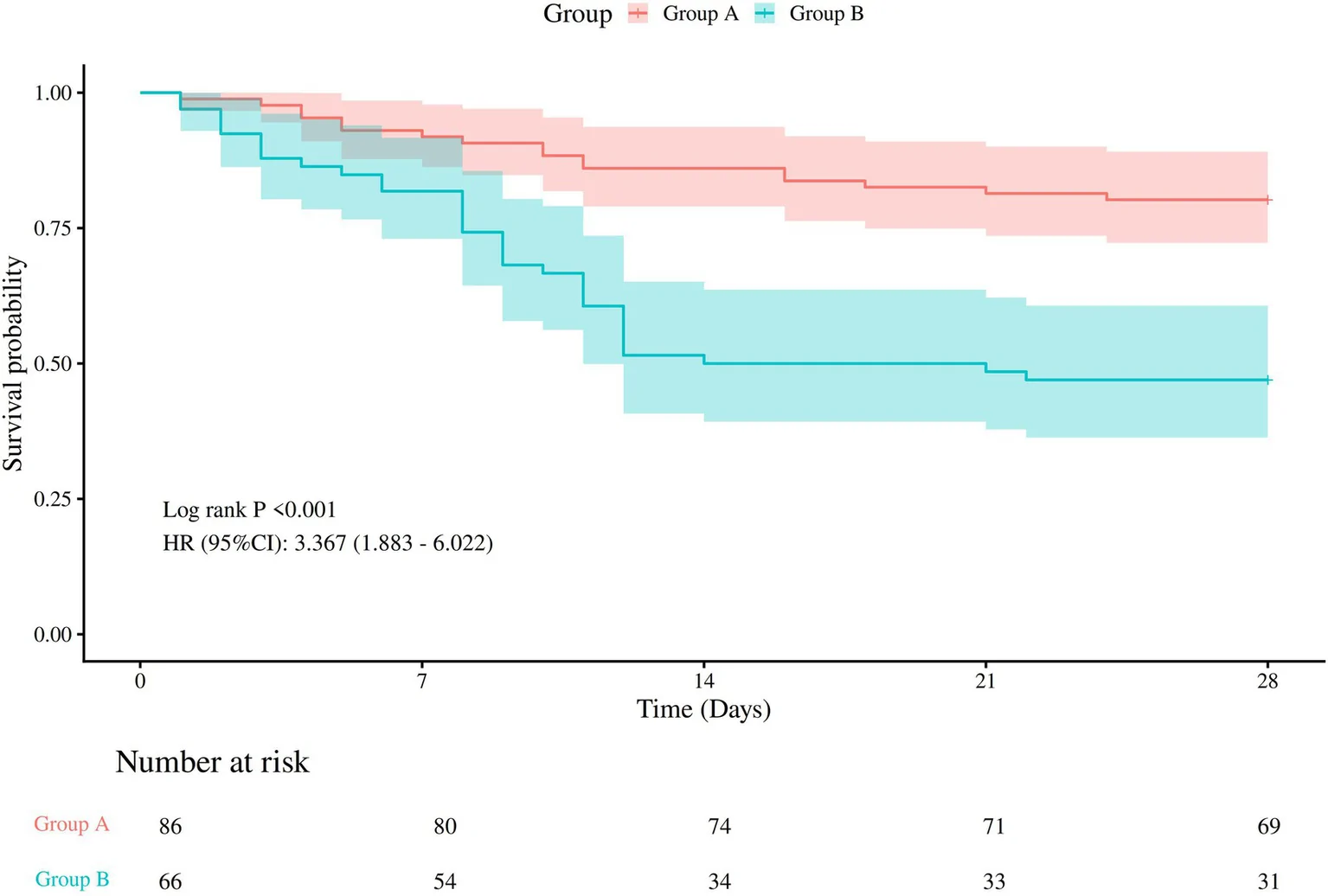
Kaplan–Meier survival curves for NUTRIC score stratification. The NUTRIC score in Group A ≥ 6; the NUTRIC score in Group B < 6.

Kaplan–Meier survival curves were generated to compare cumulative survival probabilities between high-risk (NUTRIC ≥ 6) and low-risk (NUTRIC ≤ 5) groups based on the NUTRIC score (Figure 6). The log-rank test was applied to identify statistically significant differences in survival between the groups. The results showed a significant difference in survival rates between the two groups (log-rank test: *p* < 0.001).

Patients in the high-risk group had a significantly lower cumulative survival rate compared to those in the low-risk group. The survival curves showed a clear separation at day 14, with the high-risk group exhibiting a sharper decline in survival over time. These findings further support the association between higher NUTRIC scores and increased short-term mortality in patients with sepsis-associated ARDS.

## 4 Discussion

Sepsis-associated ARDS is one of the most common and clinically important ARDS subtypes and is generally associated with greater disease severity and poorer outcomes than many non-sepsis-related ARDS subtypes. Patients with sepsis-associated ARDS often require prolonged mechanical ventilation, have fewer ventilator-free and ICU-free days, and consume substantial ICU resources. Accurate early identification of high-risk patients with sepsis-associated ARDS is therefore important for prognostic assessment, clinical prioritization, and timely optimization of supportive care. Nutritional risk is closely linked to immune dysregulation, metabolic stress, organ recovery, and clinical outcomes in critically ill patients. The NUTRIC score was specifically developed to identify ICU patients at high nutritional risk and at increased risk of adverse outcomes potentially modifiable by nutritional therapy. Although the NUTRIC score has been evaluated in general ICU populations and in patients with sepsis, evidence regarding its prognostic value in the specific population of patients with sepsis-associated ARDS remains limited. In this study, we evaluated the association between the NUTRIC score and 28-day mortality in patients with sepsis-associated ARDS using progressively adjusted multivariable models, together with RCS, ROC, calibration curve, decision curve, survival analyses and subgroup and interaction analyses.

Across all four progressively adjusted multivariable models, higher NUTRIC scores were consistently associated with increased odds of 28-day mortality. In the final adjusted model, for every one-unit increase in the NUTRIC score, each one-unit increase in the NUTRIC score was associated with 65% higher odds of death (OR = 1.65, 95% CI: 1.34–2.02, *p* < 0.001). Cox proportional hazards regression yielded concordant results, with a fully adjusted HR of 1.41 (95% CI: 1.24–1.61, *p* < 0.001). Furthermore, quartile-based analysis showed a graded increase in the odds of mortality across higher NUTRIC score categories, with a significant trend after adjustment. Restricted cubic spline analysis supported an approximately linear positive association between the NUTRIC score and the odds of 28-day mortality, with no evidence of significant nonlinearity (P for nonlinearity = 0.764). The NUTRIC score showed moderate discrimination, with an apparent AUC of 0.743 (bootstrap 95% CI, 0.661–0.827). Compared with the modified NUTRIC score (AUC 0.712), the original NUTRIC score had significantly higher discriminative performance (DeLong *p* = 0.003) and improved both reclassification (categorical NRI 0.219, *p* < 0.001) and discrimination (IDI 0.044, *p* < 0.001). In contrast, the discriminative performance of NUTRIC was comparable to that of SOFA and APACHE II (all NRI/IDI *p* > 0.05). Decision curve analysis demonstrated a positive net benefit for the NUTRIC score across threshold probabilities of 11 to 60%, supporting its utility for risk stratification. Notably, a significant interaction was observed between the NUTRIC score and septic shock status (*p* for interaction = 0.038), with the prognostic effect of NUTRIC being substantially stronger in patients with septic shock (adjusted HR 1.65) compared to those without (adjusted HR 1.28). Finally, the Kaplan–Meier analysis with the log-rank test verified that high-risk patients (NUTRIC score ≥6) had significantly lower cumulative survival than low-risk patients (*p* < 0.001). Taken together, with enhanced performance over its modified version and particular prognostic value in the presence of septic shock. These results support the use of the NUTRIC score for early mortality risk stratification in this high-risk population and extend its application to a clinically distinct ARDS phenotype characterized by systemic inflammation, respiratory failure, and nutritional vulnerability.

The observed association between the NUTRIC score and mortality may be explained, at least in part, by the profound metabolic disturbances that accompany sepsis-associated critical illness. Previous studies have evaluated the NUTRIC or mNUTRIC score in general ICU, sepsis (14), pneumonia (13), postoperative (15), and COVID-19 populations (16); our study adds phenotype-specific evidence by focusing on patients with sepsis-associated ARDS. This may be attributable to the unique pathophysiological characteristics of ARDS, which can amplify the impact of malnutrition. The NUTRIC score integrates several dimensions of critical illness, including age, disease severity, organ dysfunction, comorbidity burden, delayed ICU admission, and systemic inflammation, thereby reflecting both nutritional risk and overall physiological reserve. Under stress, the body’s metabolism undergoes profound reprogramming. The acute phase is marked by enhanced glycolysis (17, 18), insulin resistance, increased protein catabolism, and impaired lipid breakdown and utilization (19). During the recovery phase, metabolism gradually shifts toward an anabolic state (17), with lipids becoming an increasingly important energy source (20). Patients with higher NUTRIC scores generally have greater disease severity, higher inflammatory burden, and more pronounced metabolic stress, which may identify a subgroup with greater vulnerability to underfeeding and greater potential need for individualized nutritional support. Clinical studies suggest that patients with high nutritional risk may be more likely to benefit from adequate protein-energy delivery, although the optimal timing, dose, and composition of nutritional therapy in sepsis-associated ARDS require further prospective evaluation (21).

The activation and dysregulation of multiple overlapping and interacting injury pathways, along with inflammation and coagulation in both the lungs and the systemic circulation, are key pathological mechanisms of ARDS (22). Although these pathways are initially part of the host defense response to infection or injury, excessive or persistent activation can become maladaptive and contribute to progressive lung injury and organ failure (22). Both pulmonary and extra-pulmonary factors activate inflammatory cells to produce large amounts of inflammatory mediators, leading to damage of the epithelial and endothelial barriers. Protein-rich pulmonary edema leaks from the vasculature into the airspaces, further exacerbating ARDS in sepsis patients. The extent of pulmonary injury, systemic inflammation, and organ dysfunction differs substantially among patients, contributing to the clinical and biological heterogeneity of ARDS and supporting the need for phenotype-specific risk assessment. Within this context, the NUTRIC score may be clinically meaningful not merely as a nutritional screening tool, but as an integrated indicator of disease severity, inflammatory burden, and nutritional vulnerability in sepsis-associated ARDS.

The NUTRIC score demonstrated moderate discrimination in our cohort (AUC, 0.743), which was lower than that reported for mNUTRIC by Wełna et al. in 146 ICU patients with sepsis (AUC, 0.833) and by Toscano et al. in 143 medical ward patients with sepsis (AUC, 0.814) (14, 23). These differences may reflect variations in cohort characteristics, outcome definitions, and score composition. Our study was restricted to patients with sepsis-associated ARDS, a high-risk population with relatively severe organ dysfunction and a potentially narrower spectrum of baseline risk, which may reduce the discriminative ability of a prognostic score. In addition, our study and Wełna et al. evaluated 28-day mortality, whereas Toscano et al. assessed in-hospital mortality, which may be influenced by length of stay and subsequent complications. The cohort-derived optimal cutoff was 5.5 in our study, corresponding clinically to a score of ≥6, compared with 4.5 and 6 in the two previous studies. Such variation may reflect differences in case mix, outcome definitions, score distributions, and cutoff-selection methods, underscoring the need to validate prognostic thresholds in the intended target population rather than apply them universally.

The original NUTRIC score incorporates age, APACHE II score, SOFA score, number of comorbidities, days from hospital admission to ICU admission, and IL-6 levels (24). Since IL-6 is not routinely measured early in ICU care, Rahman et al. developed a modified version (mNUTRIC) that excludes IL-6 (25). Studies have shown that mNUTRIC has similar predictive efficacy in some populations (16, 26).

However, IL-6 is a key mediator of systemic inflammation and alveolar-capillary barrier damage in sepsis-related ARDS, leading to protein-rich pulmonary edema (27). Its inclusion may therefore capture inflammatory heterogeneity that is not fully represented by conventional severity measures. In our cohort, the original NUTRIC score showed modestly better discrimination than mNUTRIC (DeLong *p* = 0.003) and improved risk reclassification (NRI, 0.219; IDI, 0.044; both *p* < 0.001). These findings suggest that IL-6 may provide incremental prognostic information in patients with sepsis-associated ARDS. The original score may therefore be preferable when IL-6 is routinely measured and rapidly available without delaying nutritional-risk assessment. However, the absolute AUC difference was modest, and mNUTRIC remains more practical in settings where IL-6 testing is unavailable, costly, selectively ordered, or delayed.

The NUTRIC score also had numerically higher AUCs than PNI and CONUT (0.743 vs. 0.622 and 0.656, respectively), although neither difference reached statistical significance (PNI, *p* = 0.057; CONUT, *p* = 0.128). Unlike PNI and CONUT, which are derived primarily from laboratory indicators, NUTRIC integrates acute disease severity, organ dysfunction, comorbidity, and inflammatory burden and may therefore provide a broader assessment of mortality risk in critically ill patients. Moreover, it can be calculated without patient-reported dietary information, which is advantageous in sedated or mechanically ventilated patients.

A notable finding was the significant interaction between the NUTRIC score and septic shock status. The association between each 1-point increase in the NUTRIC score and 28-day mortality was stronger in patients with septic shock than in those without septic shock (adjusted HR, 1.65 vs. 1.28; P for interaction = 0.038). This finding suggests that the prognostic gradient of the NUTRIC score may be more pronounced in patients with severe physiological and metabolic stress. Septic shock is characterized by systemic inflammation, circulatory and metabolic abnormalities, and extensive organ dysfunction; therefore, the NUTRIC score may more comprehensively reflect the cumulative disease burden associated with mortality in this subgroup. No significant interactions were identified for ARDS severity, infection site, or vasopressor use, although the effect estimates were generally consistent with the primary analysis. Given the limited sample size, small number of events within some strata, and multiple subgroup comparisons, the septic shock interaction should be regarded as exploratory and requires confirmation in larger cohorts.

Decision curve analysis further showed that the NUTRIC score provided greater net benefit than the treat-all and treat-none strategies across clinically relevant threshold probabilities of 11–60%, with net benefit broadly comparable to that of SOFA and APACHE II. These findings suggest that the NUTRIC score may support bedside risk stratification by identifying patients who warrant closer clinical and nutritional assessment at relevant risk thresholds. Its potential value lies in integrating acute physiological derangement, organ dysfunction, chronic disease burden, and inflammatory status into a single critical illness-specific nutrition-risk metric. From a practical perspective, patients with sepsis-associated ARDS and a high NUTRIC score, including those above the cohort-derived cutoff of 6, could be prioritized for comprehensive nutritional assessment, closer monitoring of nutritional intake and feeding tolerance, and early dietitian involvement. When clinically appropriate, nutritional support should follow established critical care principles, including early and progressive enteral nutrition after hemodynamic stabilization, individualized advancement toward energy and protein targets, and assessment for refeeding risk with appropriate electrolyte monitoring (28). Nevertheless, the net benefit demonstrated by decision curve analysis reflects the potential utility of the NUTRIC score for prognostic risk stratification rather than evidence that NUTRIC-guided nutritional interventions improve clinical outcomes. This question should be addressed in randomized or rigorously controlled prospective studies. Future trials in sepsis-associated ARDS should stratify patients by NUTRIC risk and test whether early enteral nutrition, progressive energy escalation, or different protein targets produce different effects in low- and high-risk groups. Such trials would help determine whether the clinical value of NUTRIC lies only in prognostic stratification or also in identifying patients who derive greater benefit from targeted nutritional support. And, consider heterogeneity of treatment effect, future clinical trials in patients with sepsis-associated ARDS should incorporate prespecified stratification by NUTRIC risk and evaluate whether the effects of nutritional dose, timing, and protein targets differ across risk strata, thereby advancing a precision-nutrition approach in this population.

Several limitations should be acknowledged when interpreting these findings. First, this was a single-center retrospective study with a relatively small sample size, which may limit the generalizability of the findings. Although bootstrap internal validation yielded stable estimates, external validation in independent, prospectively recruited cohorts is needed. Second, because of the observational design, the association observed in this study should not be interpreted as causal. Residual confounding may also remain because of unmeasured or incompletely measured factors, including medication exposure, infection source and source-control measures, nutritional support practices, and mechanical ventilation strategies. Third, detailed data on nutritional exposure, including the timing and adequacy of energy and protein delivery, feeding intolerance, and interruptions, were unavailable. Therefore, we could not determine whether subsequent nutritional support modified the association between the NUTRIC score and mortality or whether patients with higher scores derived greater benefit from nutritional intervention. Fourth, the NUTRIC score does not directly measure all dimensions of nutritional status, such as muscle mass, body composition, micronutrient deficiencies, or longitudinal nutritional intake, all of which may influence outcomes in critically ill patients. Fifth, the limited number of events in certain subgroups, such as patients with severe ARDS or those receiving vasopressors, may have reduced the statistical power to detect interactions. Therefore, nonsignificant subgroup findings should not be interpreted as evidence of no heterogeneity. Sixth, use of the original NUTRIC score requires measurement of IL-6. If IL-6 testing is not routinely performed, restricting inclusion to patients with available IL-6 measurements may introduce selection bias and reduce the feasibility of applying the original score. In such settings, the mNUTRIC score may represent a more practical alternative. Despite these limitations, our study contributes to early risk stratification in patients with sepsis-associated ARDS by highlighting the prognostic relevance of a critical illness-specific nutritional risk score in a population characterized by inflammation, hypoxemia, organ dysfunction, and high metabolic demand.

## 5 Conclusion

In patients with sepsis-associated ARDS, a higher NUTRIC score was independently associated with increased 28-day mortality and showed clinically meaningful performance for early risk stratification. Beyond reflecting nutritional risk alone, the NUTRIC score may serve as an integrated indicator of inflammatory burden, organ dysfunction, and metabolic stress in this highly heterogeneous critically ill population. The original NUTRIC score incorporating IL-6 outperformed the modified version in both discrimination and risk reclassification, supporting the incremental prognostic value of this inflammatory biomarker in this population. The prognostic effect was amplified in patients with septic shock, suggesting that the NUTRIC score may be particularly informative in those with the highest disease severity. With moderate discrimination, excellent calibration, and a positive net benefit across clinically relevant threshold probabilities, the NUTRIC score represents a practical, integrative tool for early mortality risk stratification in sepsis-associated ARDS. These findings support the incorporation of NUTRIC-based assessment into the early ICU evaluation framework for patients with sepsis-associated ARDS. Future studies should validate these findings in larger, multicenter prospective cohorts and determine whether risk-adapted nutritional strategies based on NUTRIC-defined risk profiles can improve clinically meaningful outcomes in patients with sepsis-associated ARDS.

## Data Availability

The raw data supporting the conclusions of this article will be made available by the authors, without undue reservation.
